# Oncological outcome after robot-assisted *versus* open pancreatoduodenectomy for upfront resectable cancer in the pancreatic head: a nationwide cohort analysis

**DOI:** 10.1093/bjs/znaf153

**Published:** 2025-11-28

**Authors:** Julia E Menso, Caro L Bruna, Mahsoem Ali, Bert Bonsing, Koop Bosscha, Lodewijk A A Brosens, Olivier R Busch, A Stijn L P Crobach, Freek Daams, Wouter Derksen, Maxime J L Dewulf, Michail Doukas, Arantza Fariña Sarasqueta, Sebastiaan Festen, Mohammad Abu Hilal, Ignace H J T de Hingh, Marjolein Y V Homs, Geert Kazemier, Daan J Lips, Misha D P Luyer, Vincent E de Meijer, J Sven D Mieog, Wouter W te Riele, Hjalmar C van Santvoort, George P van der Schelling, Martijn Stommel, Joanne Verheij, Roeland F de Wilde, Johanna W Wilmink, I Quintus Molenaar, Bas Groot Koerkamp, Lydia G van der Geest, Marc G Besselink

**Affiliations:** Department of Surgery, Amsterdam UMC, location University of Amsterdam, Amsterdam, The Netherlands; Cancer Center Amsterdam, Amsterdam, The Netherlands; Department of Surgery, Amsterdam UMC, location University of Amsterdam, Amsterdam, The Netherlands; Cancer Center Amsterdam, Amsterdam, The Netherlands; Cancer Center Amsterdam, Amsterdam, The Netherlands; Department of Surgery, Amsterdam UMC, location Vrije Universiteit, Amsterdam, The Netherlands; Department of Surgery, Leiden University Medical Center, Leiden, The Netherlands; Department of Surgery, Jeroen Bosch Hospital, ‘s-Hertogenbosch, The Netherlands; Department of Pathology, Regional Academic Cancer Center Utrecht (RAKU), UMC Utrecht, Utrecht, The Netherlands; Department of Surgery, Amsterdam UMC, location University of Amsterdam, Amsterdam, The Netherlands; Cancer Center Amsterdam, Amsterdam, The Netherlands; Department of Pathology, Leiden University Medical Center, Leiden, The Netherlands; Cancer Center Amsterdam, Amsterdam, The Netherlands; Department of Surgery, Amsterdam UMC, location Vrije Universiteit, Amsterdam, The Netherlands; Department of Surgery, Regional Academic Cancer Center Utrecht (RAKU), UMC Utrecht, Utrecht, The Netherlands; Department of Surgery, Maastricht University Medical Center, Maastricht, The Netherlands; Department of Pathology, Erasmus MC, Rotterdam, The Netherlands; Cancer Center Amsterdam, Amsterdam, The Netherlands; Department of Pathology, Amsterdam UMC, location University of Amsterdam, Amsterdam, The Netherlands; Department of Surgery, OLVG, Amsterdam, The Netherlands; Department of Surgery, Amsterdam UMC, location University of Amsterdam, Amsterdam, The Netherlands; Department of Surgery, School of Medicine, University of Jordan, Amman, Jordan; Department of Surgery, University Hospital Southampton NHS Foundation Trust, Southampton, UK; Department of Surgery, Catharina Hospital, Eindhoven, The Netherlands; Department of Medical Oncology, Erasmus MC Cancer Institute, Rotterdam, The Netherlands; Cancer Center Amsterdam, Amsterdam, The Netherlands; Department of Surgery, Amsterdam UMC, location Vrije Universiteit, Amsterdam, The Netherlands; Department of Surgery, Medisch Spectrum Twente, Enschede, The Netherlands; Department of Surgery, Catharina Hospital, Eindhoven, The Netherlands; Department of Surgery, University of Groningen and University Medical Center Groningen, Groningen, The Netherlands; Department of Surgery, Leiden University Medical Center, Leiden, The Netherlands; Department of Surgery, Regional Academic Cancer Center Utrecht (RAKU), UMC Utrecht, Utrecht, The Netherlands; Department of Surgery, Regional Academic Cancer Center Utrecht (RAKU), UMC Utrecht, Utrecht, The Netherlands; Department of Surgery, Amphia Hospital, Breda, The Netherlands; Department of Surgery, Radboud University Medical Center, Nijmegen, The Netherlands; Cancer Center Amsterdam, Amsterdam, The Netherlands; Department of Pathology, Amsterdam UMC, location University of Amsterdam, Amsterdam, The Netherlands; Department of Surgery, Erasmus MC Cancer Institute, Rotterdam, The Netherlands; Cancer Center Amsterdam, Amsterdam, The Netherlands; Department of Medical Oncology, Amsterdam UMC, location University of Amsterdam, Amsterdam, The Netherlands; Department of Surgery, Regional Academic Cancer Center Utrecht (RAKU), UMC Utrecht, Utrecht, The Netherlands; Department of Surgery, Erasmus MC Cancer Institute, Rotterdam, The Netherlands; Department of Research and Development, Netherlands Comprehensive Cancer Organisation (IKNL), Utrecht, The Netherlands; Department of Surgery, Amsterdam UMC, location University of Amsterdam, Amsterdam, The Netherlands; Cancer Center Amsterdam, Amsterdam, The Netherlands

## Abstract

**Background:**

Robot-assisted pancreatoduodenectomy (RPD) aims to enhance postoperative recovery compared to open pancreatoduodenectomy (OPD). Although recent randomized trials confirmed the short-term safety of RPD, they did not confirm superiority or assess oncological safety. This nationwide observational cohort study compares oncological outcome after RPD *versus* OPD in patients with resectable pancreatic ductal adenocarcinoma (PDAC) and distal cholangiocarcinoma (DCC) without vascular contact.

**Methods:**

All consecutive patients undergoing RPD and OPD for upfront resectable PDAC and DCC without vascular contact on preoperative imaging in the Netherlands were included. Data were obtained from the Netherlands Cancer Registry (2016–2023). Primary outcomes were overall survival (OS) and R0-resection rate.

**Results:**

Overall, 1675 patients after pancreatoduodenectomy for upfront resectable PDAC and DCC were included (375 RPD; 1300 OPD). Adjusted median OS was 23 months after RPD *versus* 22 months after OPD, with comparable 5-year survival rate (23.3% *versus* 22.4%, HR 0.96 [0.82–1.14], *P* = 0.665). The R0-resection rate was comparable (57.1% *versus* 59.7%, *P* = 0.368). RPD was associated with a shorter hospital stay (median 9 *versus* 11 days, *P* < 0.001) and comparable in-hospital/30-day (3.1% *versus* 2.6%, *P* = 0.618) and 90-day mortality rate (7.7% *versus* 6.2%, *P* = 0.276). In patients with PDAC, no differences in receipt (58.2% *versus* 58.7%, *P* = 0.900), time to start (median 54 *versus* 58 days, *P* = 0.107), or completion of adjuvant chemotherapy (30.4% *versus* 30.4%, *P* = 0.999) were observed.

**Conclusions:**

In this nationwide study, oncological outcome including 5-year survival was comparable between patients undergoing RPD and OPD for upfront resectable PDAC and DCC without vascular contact without differences in the use of adjuvant therapy for PDAC.

## Teaser

In this nationwide registry-based study, the oncological outcome including 5-year survival was comparable between patients undergoing robot-assisted and open pancreatoduodenectomy for primary resectable pancreatic ductal adenocarcinoma and distal cholangiocarcinoma without vascular contact.

## Introduction

Pancreatic ductal adenocarcinoma (PDAC) and distal cholangiocarcinoma (DCC) remain among the most fatal cancers^1^. Patients diagnosed with resectable cancer in the pancreatic head have a survival of 14–33 months after diagnosis in all patients with stage I–II disease^2–4^. The only potentially curative treatment for these patients is pancreatoduodenectomy, which carries a high risk of complications, hindering postoperative recovery and therefore potentially impeding start and completion of adjuvant therapy^5,6^.

Robot-assisted pancreatoduodenectomy (RPD) aims to improve postoperative recovery compared to the traditional open approach (OPD)^7,8^ . Faster and enhanced recovery after RPD might facilitate a more rapid initiation and endurance of adjuvant chemotherapy, potentially impacting survival of patients with cancer in the pancreatic head. In addition, RPD may also reduce the impact of surgical therapy on the host’s immune system. Even after uncomplicated major abdominal surgery, postoperative fatigue due to postsurgical inflammation could last up to 3 months after resection, potentially delaying the start of adjuvant therapy^9–11^.

However, concerns have been raised regarding the oncological outcome of minimally invasive major abdominal surgery. Multiple randomized controlled trials (RCTs) and meta-analyses for cervical cancer and rectal cancer could not establish the non-inferiority of the minimally invasive approach in terms of disease-free survival and radical resection^12–16^. Two recent RCTs which compared RPD with OPD found no difference in short-term oncological outcome in the subgroup of patients with malignant disease (that is lymph node retrieval and resection radicality), but did not report on survival^17,18^. Other studies about oncological outcome after RPD and OPD for PDAC and DCC mostly consist of single-centre retrospective series and might not be generalizable to other patients and centres^19,20^.

This nationwide study aimed to evaluate oncological outcome following RPD *versus* OPD in patients with upfront resectable cancer in the pancreatic head without vascular contact.

## Methods

### Study design

The current study was a nationwide observational cohort study, including consecutive patients with suspected or proven upfront resectable cancer in the pancreatic head (that is PDAC and DCC) without vascular contact undergoing RPD and OPD in the Netherlands (2016–2023). This study followed the STROBE guidelines for reporting observational studies (*Table S1*).

### Study ethics

Ethical approval was granted by the Medical Ethics Committee of the Amsterdam UMC on 24 July 2024.

### Data collection

Data were retrieved from the nationwide database of the Netherlands Cancer Registry (NCR), maintained by the Netherlands Comprehensive Cancer Organization (in Dutch: IKNL). Cancer diagnoses are notified to the NCR by the nationwide pathology database (Palga) and the Dutch National Hospital Care Registration (hospital discharges and outpatient visits). Independent IKNL registration personnel verify all notifications in patient files in all Dutch hospitals approximately 9–12 months after diagnosis and record additional data on patient, tumour, and treatment characteristics. Dates of death or emigration were added by linkage with the nationwide Municipal Personal Records Database (last update 1 February 2024). The accuracy of the NCR is evaluated by internal audits and feedback from users (‘living database’).

### Eligibility

Adult patients (≥18 years old) undergoing RPD and OPD for upfront resectable PDAC and DCC (including adenocarcinoma from precursor lesions) without vascular contact on imaging before treatment (International Classification of Diseases for Oncology 4th edition topography C25 pancreas [excluding C25.4] and C24.2 distal bile duct with morphology adenocarcinoma) were included (*Table S2*). In the present study, both PDAC and DCC were included because preoperative differentiation can be challenging. Resectable PDAC and DCC are defined as <90 degrees venous involvement, according to the criteria of the Dutch Pancreatic Cancer Group^21^. However, only patients without vascular involvement (also no venous contact) on preoperative imaging are currently eligible for RPD in the majority of centres in Netherlands. Therefore, in this study, patients undergoing OPD were included based on the RPD selection criteria to minimize selection bias: no vascular contact on preoperative imaging, BMI <35 kg/m^2^, and no distant metastases (including distant lymph nodes). The BMI criterion was not applied for the selection of OPD patients in this study due to missing data. Preoperative carbohydrate antigen 19.9 (CA19.9) and carcinoembryonic antigen (CEA) levels were used solely for prognostic purposes and were not considered in the determination of tumour resectability.

Patients with vascular involvement on preoperative imaging and patients undergoing laparoscopic pancreatoduodenectomy were excluded. Following concerning mortality outcomes and the lack of patient benefits in the LEOPARD-2 trial comparing laparoscopic with open pancreatoduodenectomy, the use of laparoscopic pancreatoduodenectomy was discontinued in the Netherlands^22^.

### Outcomes

The primary outcomes were overall survival (OS, median, years) and R0-resection rate^23–25^. Secondary outcomes were receipt and type of adjuvant therapy, time to start adjuvant therapy (days), number of cycles and completion of adjuvant therapy, lymph node yield, positive lymph node ratio (number of positive lymph nodes divided by number of yielded lymph nodes), and 1-/3-/5-year survival.

Baseline variables included age (years), sex, ASA score, and co-morbidities. Other pretreatment variables included laboratory results, modified Glasgow Prognostic score (mGPS), tumour size on imaging before treatment (mm), and receipt of neoadjuvant treatment. Intraoperative variables included year of surgery, conversion, and vascular resection. Pathological variables included final pathological diagnosis, tumour size (mm), differentiation grade, yielded and positive lymph nodes, and radical (R0) resection. Postoperative outcomes included length of hospital stay (days), in-hospital/30-day and 90-day mortality rate, and oncological outcomes including receipt of adjuvant therapy.

### Definitions

Co-morbidities were scored according to the Charlson Co-morbidity Index (CCI), and included malignancy, myocardial infarction, cerebrovascular disease, diabetes mellitus, renal insufficiency, and liver disease^26,27^. Modified Glasgow Prognostic score is based on C-reactive protein (CRP) and albumin levels. An elevated mGPS is correlated with poorer OS^28,29^. A CRP ≥10 mg/l and albumin <35 g/l gives an mGPS of 2; CRP ≥10 mg/l and albumin ≥35 g/l gives an mGPS of 1; and a normal CRP of <10 mg/l results in an mGPS of 0, regardless of albumin levels. Conversion to OPD was defined as a robot-assisted procedure in which an incision was made for other reasons than trocar placement or specimen extraction. Venous resection included resection of the superior mesenteric and/or portal vein. Arterial resection included resection of the common hepatic artery, superior mesenteric artery, and/or the celiac trunk. R0-resection was defined according to the Royal College of Pathologists definition and the Dutch guidelines as achieving a tumour-free margin of ≥1 mm for all margins and surfaces (except for the anterior surface, where a tumour-free margin of <1 mm is acceptable), R1-resection indicated microscopic tumour involvement in one of the margins/surfaces, and R2-resection indicated macroscopically remaining tumour after resection. Overall survival was calculated as the interval between date of resection and date of death or end of follow-up.

### Surgical technique

The International Study Group for Pancreatic Surgery (ISGPS) standard for lymphadenectomy was used^30^. Dissection in both RPD and OPD closely follows the superior mesenteric artery, but this artery typically is not fully skeletonized. Pylorus resection or preservation was to the local surgeons’ discretion. Since 2021, several centres implemented the use of the teres ligament as patch between the pancreaticojejunostomy and the gastroduodeneal artery stump to prevent post-pancreatectomy hemorrhage^31^. For OPD, an epidural or preperitoneal wound catheters are commonly placed^32^.

### Postoperative management

In the Netherlands, postoperative management for patients undergoing pancreatoduodenectomy is provided according to the Enhanced Recovery After Surgery protocol since 2010^33^. Additionally, the new best practice algorithm of the PORSCH-trial was implemented between 2018 and 2019 for standardization of postoperative care in patients undergoing pancreatic surgery, resulting in a decreased mortality rate^34^. The centres that perform both RPD and OPD provide similar postoperative care for both approaches.

### Oncological treatment

In the Netherlands, the PREOPANC, PREOPANC-2, and PREOPANC-3 RCTs included patients with resectable PDAC during the study period of the current study. The majority of patients with resectable PDAC who received neoadjuvant FOLFIRINOX did so in the ongoing PREOPANC-3 and the PREOPANC-2 randomized trial^35,36^. The majority of patients with resectable PDAC who received neoadjuvant chemoradiation did so in the PREOPANC-2 or PREOPANC trial^36^. Neoadjuvant therapy for patients with resectable PDAC is currently not incorporated in the national guidelines. All patients with a final pathological diagnosis of PDAC are advised to receive adjuvant therapy. Patients with a WHO performance score <2 and up to 75 years old receive modified FOLFIRINOX (mFOLFIRINOX), whereas the other patients receive gemcitabine(–capecitabine)^37–39^. In the Netherlands, patients with resected DCC as final pathological diagnosis are not advised to receive adjuvant therapy.

### Statistical analysis

Data were analysed with IBM SPSS Statistics version 28 for Windows (descriptives), R-studio version 4.3.2 (Cox regression analyses), and Stata version 18 (flexible parametric survival models and regression standardization, using the stmp3 module^40^).

Continuous data were analysed with the Mann–Whitney U test and presented as medians with interquartile ranges (i.q.r.s) presented here as 25th and 75th percentiles. Categorical data were analysed with Pearson’s chi-squared test and presented as frequencies with percentages.

Multivariable Cox regression was used for the association of RPD *versus* OPD with OS, with adjustment for the following prespecified confounders, which were identified based on the literature and using directed acyclic graphs: age at diagnosis, sex, ASA score, CCI score, diabetes mellitus, highest CA19.9 level before treatment (log-transformed), mGPS, tumour diameter on imaging before treatment, year of surgery, positive lymph node ratio (number of positive lymph nodes divided by number of yielded lymph nodes), tumour differentiation grade, and neoadjuvant chemo(radio)therapy in PDAC^29,41–43^. Continuous variables were modelled with restricted cubic splines (three knots) to capture potential nonlinear covariate–outcome relationships and prevent residual confounding. Flexible parametric survival models followed by regression standardization were used to estimate the adjusted median/1-/3-/5-year survival estimates, adjusted for the same confounders mentioned previously. The proportional hazards assumption was checked using visual inspection of Schoenfeld residuals and the Grambsch–Therneau test. Survival estimates were presented as frequencies with 95% confidence intervals.

Missing data were handled using flexible multiple imputation models that were congenial with the main analysis model. The imputation model contained the treatment variable (RPD *versus* OPD), all potential confounders, the event variable, and the Nelson–Aalen estimate of the cumulative baseline hazard. In total, 50 imputed data sets were generated after 50 burn-in iterations.

Subgroup analyses were performed for PDAC with and without administration of neoadjuvant therapy (NAT), which can potentially affect the primary outcome OS, and for DCC, because of small differences in prognosis as compared to PDAC^44^. As (neo)adjuvant therapy for patients with DCC is not recognized in the Netherlands, the analyses for (neo)adjuvant therapy included patients with PDAC only. Finally, interaction analyses were performed to identify patient subgroups that benefit most from RPD and OPD. The subgroups included sex, age (<65 years or ≥65 years), ASA score (I–II or III–IV), tumour size (<20 mm, 20–40 mm, or >40 mm), CA19.9 level (<37, 37–150, 150–500, or >500), pathological diagnosis (PDAC or DCC), NAT, and mGPS^29,45^. Interaction values of *P* were obtained using likelihood ratio tests. As RPD was implemented in the Netherlands in 2018, a sensitivity analysis was performed to assess the impact of potential time bias and RPD without standardized training by excluding all patients undergoing RPD and OPD before 2018.

A two-tailed *P* < 0.050 was considered statistically significant.

## Results

In total, 1675 patients with resected PDAC and DCC were included (375 RPD and 1300 OPD) from all 15 centres for pancreatic surgery in the Netherlands. Robot-assisted pancreatoduodenectomy was officially introduced in the Netherlands in 2018 (*Fig. S1*).

The median age was 69 years and 41.7% of patients were female. No significant differences in patient characteristics were found between groups (*Table 1*), except for a smaller tumour size on preoperative imaging (22 *versus* 24 mm, *P* < 0.001) and more patients with PDAC having neoadjuvant therapy in the robotic group (19.7% *versus* 11.6%, *P* = 0.002).

**Table 1:** Patient characteristics and preoperative treatment in patients undergoing robot-assisted *versus* open pancreatoduodenectomy

Patient characteristics	Total (*n* = 1675)	RPD (*n* = 375)	OPD (*n* = 1300)	*P*
Age (years)	69 (62–75)	70 (63–75)	69 (62–75)	0.572
Female	699 (41.7%)	152 (40.5%)	547 (42.1%)	0.593
**ASA score ≥3**	592 (36.1%)	128 (34.9%)	464 (36.4%)	0.581
Missing	35	8	27	
CCI ≥2*	300 (18.7%)	65 (17.7%)	235 (19.0%)	0.566
Missing	74	8	66	
**Diabetes mellitus**	345 (21.5%)	75 (20.4%)	270 (21.9%)	0.555
Missing	74	8	66	
Preoperative diagnostics*				
CA19-9 (U/ml)	157 (46–552)	162 (51–497)	157 (44–574)	0.850
**<37 U/ml**	302 (21.7%)	66 (20.6%)	236 (22.0%)	0.602
Missing	282	55	227	
**37–499 U/ml**	720 (51.5%)	174 (53.9%)	546 (50.8%)	0.332
Missing	277	52	225	
**≥500 U/ml**	371 (26.6%)	80 (25.0%)	291 (27.1%)	0.451
Missing	282	55	227	
Total bilirubin (µmol/l)	173 (72–267)	177 (67–252)	173 (75–270)	0.473
Albumin (g/l)	35 (30–40)	35 (31–40)	36 (30–40)	0.697
CRP (mg/l)	12 (5–32)	12 (5–33)	12 (6–32)	0.739
**mGPS**				0.948
0	547 (49.1%)	116 (48.7%)	431 (49.1%)	
1	249 (22.3%)	52 (21.8%)	197 (22.5%)	
2	319 (28.6%)	70 (29.4%)	249 (28.4%)	
Missing	560	137	423	
Tumour size (mm)	24 (18–30)	22 (17–28)	24 (19–30)	<0.001
**Preoperative treatment**†				
Neoadjuvant therapy	134 (13.3%)	41 (19.7%)	93 (11.6%)	0.002
Chemotherapy	83 (61.9%)	24 (58.5%)	59 (63.4%)	0.686
Radiotherapy	5 (3.7%)	1 (2.4%)	4 (4.3%)	N/A
Chemoradiation	46 (34.3%)	16 (39.0%)	30 (32.3%)	N/A
Number of cycles	6 (5–7)	7 (3–7)	6 (5–8)	0.773
Completion	33 (38.8%)	6 (26.1%)	27 (43.5%)	0.142

Values are presented in medians with interquartile ranges or frequencies with percentages. RPD, robot-assisted pancreatoduodenectomy; OPD, open pancreatoduodenectomy; CCI, Charlson Co-morbidity Index; CA19-9, carbohydrate antigen 19.9; CRP, C-reactive protein; mGPS, modified Glasgow prognostic score. *CA19.9 and total bilirubin based on the highest measurement before treatment. Albumin, CRP, and tumour size based on the last measurement before treatment. †Neoadjuvant therapy in patients with PDAC only (208 RPD, 803 OPD). Neoadjuvant chemotherapy was FOLFIRINOX in all cases (23 RPD, 62 OPD). Neoadjuvant chemoradiation was gemcitabine with radiotherapy. (22) Number of cycles and completion of the initial scheme.

### Surgical and pathological outcomes

Conversion rate in the RPD group was 10.7%. Vascular resection was performed less frequently in RPD compared to OPD (5.9% *versus 13.9*%, *P* < 0.001). The R0-resection rate was comparable between groups (57.1% *versus* 59.7%, *P* = 0.368). Final diagnosis of PDAC was 56% for RPD and 62% for OPD. The tumour size was smaller in the RPD group compared to OPD (median 25 *versus* 27 mm, *P* < 0.001) and fewer lymph nodes were resected with RPD compared to OPD (median 15 *versus* 16, *P* = 0.035), but positive lymph node ratio did not differ between groups (0.11 *versus* 0.11, *P* = 0.670, *Table 2*).

**Table 2:** Surgical and pathological outcomes of patients undergoing robot-assisted *versus* open pancreatoduodenectomy

Surgical outcomes	RPD (*n* = 375)	OPD (*n* = 1300)	*P*
Conversion	40 (10.7%)	N/A	N/A
**Vascular resection**	22 (5.9%)	178 (13.9%)	<0.001
Venous	17 (77.3%)	151 (84.8%)	0.659
Arterial	2 (9.1%)	11 (6.2%)	N/A
Both	3 (13.6%)	16 (9.0%)	N/A
**Pathological outcomes**			
Final diagnosis			0.085
PDAC	208 (55.5%)	803 (61.8%)	N/A
DCC	142 (37.9%)	418 (32.2%)	N/A
Other	25 (6.7%)	79 (6.1%)	N/A
Tumour size (mm)*	25 (18–32)	27 (20–35)	<0.001
**Differentiation grade**			0.603
Good	54 (17.8%)	183 (16.9%)	N/A
Moderate	179 (59.1%)	635 (58.7%)	N/A
Poor	70 (23.1%)	258 (23.8%)	N/A
Anaplastic	0 (0.0%)	6 (0.6%)	N/A
Missing	0	218	
Yielded lymph nodes	15 (12–19)	16 (12–21)	0.035
Positive lymph nodes	2 (0–5)	2 (0–5)	0.293
Positive lymph node ratio	0.11 (0.00–0.27)	0.11 (0.00–0.29)	0.670
**R0-resection**	213 (57.1%)	772 (59.7%)	0.368
R1 (microscopic)	160 (42.9%)	510 (39.4%)	0.113†
R2 (macroscopic)	0 (0.0%)	11 (0.9%)	N/A

Values are presented in medians with interquartile ranges or frequencies with percentages. RPD, robot-assisted pancreatoduodenectomy; OPD, open pancreatoduodenectomy; PDAC, pancreatic ductal adenocarcinoma; DCC, distal cholangiocarcinoma. *Tumour size based on pathological specimen assessment. †*P* is calculated over the group variable.

### Short-term outcome and adjuvant therapy

Length of hospital stay was shorter after RPD compared to OPD (9 *versus* 11 days, *P* < 0.001). In-hospital/30-day mortality rate (3.1% *versus* 2.6%, *P* = 0.618) and 90-day mortality rate (7.7% *versus* 6.2%, *P* = 0.276) did not differ between groups. Adjuvant chemotherapy was administered in 58.2% of patients after RPD *versus* 58.7% after OPD (*P* = 0.900) with mFOLFIRINOX (74.1% *versus* 66.7%) and gemcitabine–capecitabine (25.9% *versus* 30.3%, *P* = 0.184). Time to start (median 54 *versus* 58 days, *P* = 0.107) and completion of adjuvant chemotherapy (30.4% *versus* 30.4%, *P* = 0.999) did not differ significantly between the groups. The number of cycles of mFOLFIRINOX (median 9 *versus* 8 cycles, *P* = 0.508) and gemcitabine–capecitabine (median 5 *versus* 5 cycles, *P* = 0.219) also did not significantly differ between the groups. The proportion of patients who switched type of adjuvant chemotherapy was comparable between groups (1.0% *versus* 1.7%, *P* = 0.421), whereas more patients received dose reduction after RPD (27.3% *versus* 14.6%, *P* = 0.001, *Table 3*).

**Table 3:** Short-term outcome and adjuvant therapy in patients undergoing robot-assisted *versus* open pancreatoduodenectomy

Short-term outcomes	RPD (*n* = 375)	OPD (*n* = 1300)	*P*
Length of hospital stay (days)	9 (7–16)	11 (8–18)	<0.001
In-hospital/30-day mortality	11 (3.1%)	32 (2.6%)	0.618
90-day mortality	29 (7.7%)	80 (6.2%)	0.276
Adjuvant therapy*			
Adjuvant chemotherapy	121 (58.2%)	471 (58.7%)	0.900
mFOLFIRINOX	60 (74.1%)	198 (66.7%)	0.184
Gemcitabine-capecitabine	21 (25.9%)	90 (30.3%)	N/A
Other†	0 (0.0%)	9 (3.0%)	N/A
Missing	127	506	
Time to start chemotherapy (days)	54 (44–69)	58 (48–70)	0.107
**Number of cycles**			N/A
mFOLFIRINOX	9 (3–12)	8 (2–12)	0.508
Gemcitabine–capecitabine	5 (3–6)	5 (3–6)	0.219
**Completion**	24 (30.4%)	89 (30.4%)	0.999
Missing	129	510	
Switch	2 (1.0%)	14 (1.7%)	0.421
**Dose reduction**	33 (27.3%)	69 (14.6%)	0.001
Missing	87	332	

Values are presented in medians with interquartile ranges or frequencies with percentages. RPD, robot-assisted pancreatoduodenectomy; OPD, open pancreatoduodenectomy. *Adjuvant therapy in patients with PDAC only (208 RPD, 803 OPD). Number of cycles and completion of the initial scheme (including neoadjuvant chemotherapy). †Other types of chemotherapy included capecitabin, FOLFIRI, gemcitabin–cisplatin, irinotecan–oxaliplatin, gemcitabine–carboplatin, CAPOX, capecitabine–mitomycin, and study-related regimens.

### Survival

During follow-up, 1049 patients died (62.6%). The median follow-up was 46 months (i.q.r. 24–67). The median OS was 24 months (95% c.i., 22 to 29) after RPD *versus* 23 months (95% c.i., 21 to 24) after OPD (HR 0.93; 95% c.i., 0.79 to 1.08; *P* = 0.312), with comparable 1-year (72.8% *versus* 72.5%), 3-year (35.9% *versus* 33.5%), and 5-year survival rates (27.3% *versus* 21.7%) between groups (*Fig. S2*).

**Fig. 1:**
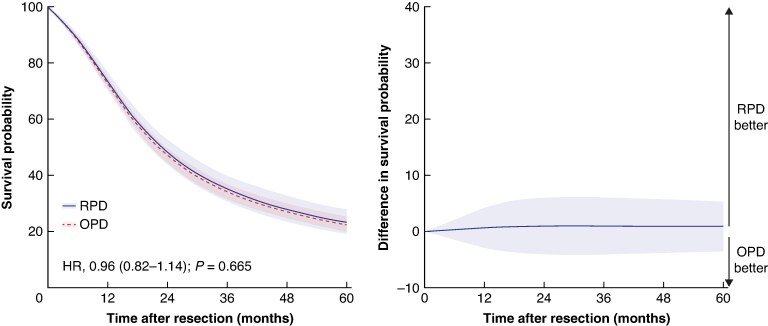
Adjusted overall survival estimates in patients undergoing robot-assisted *versus* open pancreatoduodenectomy Adjusted overall survival per surgical approach (left) and difference in overall survival between approaches (right). RPD, robot-assisted pancreatoduodenectomy; OPD, open pancreatoduodenectomy.

After adjusting for potential confounders, the median OS was 23 months (95% c.i., 20 to 26) after RPD *versus* 22 months (95% c.i., 21 to 24) after OPD (adjusted HR 0.96; 95% c.i., 0.82 to 1.14; *P* = 0.665) with comparable 1-year (73.4% *versus* 72.7%), 3-year (35.2% *versus* 34.2%), and 5-year survival rates (23.3% *versus* 22.4%) between groups (*Fig. 1* and *Table S3* for the adjusted and unadjusted survival analyses respectively). The sensitivity analysis excluding patients undergoing RPD and OPD before 2018 showed no impact with an adjusted median OS of 23 months (95% c.i., 20 to 27) after RPD *versus* 23 months (95% c.i., 21 to 25) after OPD (adjusted HR 0.96; 95% c.i., 0.80 to 1.14; *P* = 0.622).

The median OS in patients with PDAC (*n* = 1011) was 22 months (95% c.i., 19 to 25) after RPD *versus* 20 months (95% c.i., 19 to 22) after OPD (adjusted HR 0.92; 95% c.i., 0.75 to 1.13; *P* = 0.445) with comparable 1-year (71.9% *versus* 70.7%), 3-year (32.8% *versus* 31.1%), and 5-year survival rates (21.3% *versus* 19.8%) between groups (*Table 4*). In the subgroup of patients with PDAC who received NAT, no differences were found in median OS (26 *versus* 21 months; adjusted HR 0.80; 95% c.i., 0.43 to 1.47; *P* = 0.477), and 1-year (74.5% *versus* 70.7%), 3-year (43.4% *versus* 38.3%), and 5-year survival rates (35.2% *versus* 30.3%) between RPD and OPD. Also, in the subgroup of patients with PDAC without NAT, no differences were found in median OS (23 *versus* 22 months; adjusted HR 0.96; 95% c.i., 0.77 to 1.21; *P* = 0.743), and 1-year (71.8% *versus* 70.5%), 3-year (32.0% *versus* 30.3%), and 5-year survival rates (20.2% *versus* 18.8%) between groups (*Table S4*).

**Table 4:** Adjusted survival of patients undergoing robot-assisted *versus* open pancreatoduodenectomy

Survival (adjusted)	RPD (*n* = 375)	OPD (*n* = 1300)	HR (95% c.i.)	*P*
**Overall survival (months)**	23 (20–26)	22 (21–24)	0.96 (0.82–1.14)	0.665
1-year survival	73.4% (70.0–76.6)	72.7% (70.8–74.6)	N/A	N/A
3-year survival	35.2% (30.7–39.9)	34.2% (31.6–36.8)	N/A	N/A
5-year survival	23.3% (19.2–27.9)	22.4% (19.9–25.2)	N/A	N/A
**Survival PDAC (months)**	22 (19–25)	20 (19–22)	0.92 (0.75–1.13)	0.445*
1-year survival	71.9% (67.4–76.0)	70.7% (68.1–73.1)	N/A	N/A
3-year survival	32.8% (27.2–38.9)	31.1% (28.1–34.2)	N/A	N/A
5-year survival	21.3% (16.4–27.1)	19.8% (16.9–23.1)	N/A	N/A
**Survival DCC (months)**	25 (21–31)	28 (24–32)	1.05 (0.80–1.38)	0.726*
1-year survival	76.4% (71.2–81.0)	77.0% (73.6–80.0)	N/A	N/A
3-year survival	39.8% (32.6–47.4)	40.7% (35.9–45.6)	N/A	N/A
5-year survival	26.8% (20.0–34.8)	27.6% (22.7–33.1)	N/A	N/A

Values are presented in medians with interquartile ranges or percentages with 95% confidence intervals. RPD, robot-assisted pancreatoduodenectomy; OPD, open pancreatoduodenectomy; HR, Hazard ratio; CI, confidence interval; PDAC, pancreatic ductal adenocarcinoma; DCC, distal cholangiocarcinoma. *For subgroup analyses, only interaction values of *P* are calculated.

The median OS in patients with DCC (*n* = 560) was 25 months (95% c.i., 21 to 31) after RPD *versus* 28 months (95% c.i., 24 to 32) after OPD (adjusted HR 1.05; 95% c.i., 0.80 to 1.38; *P* = 0.726) with comparable 1-year (76.4% *versus* 77.0%), 3-year (39.8% *versus* 40.7%), and 5-year survival rates (26.8% *versus* 27.6%) between groups (*Table 4*).

### Predictors for overall survival

**Fig. 2:**
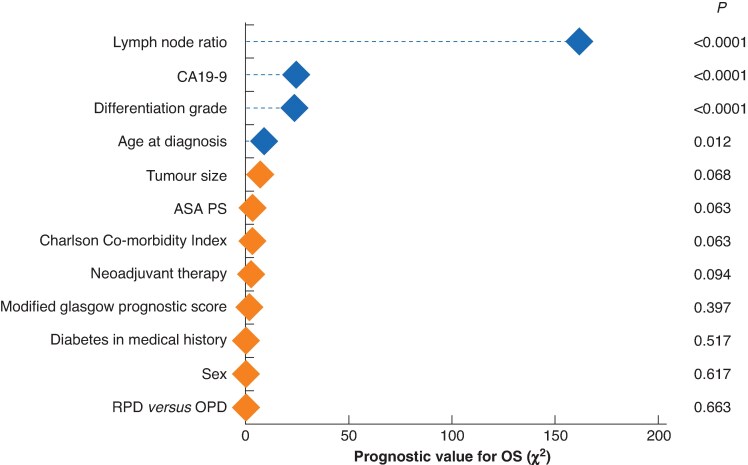
Predictors for overall survival in the multivariable model The *x*-axis represents the independent prognostic value per variable for overall survival in a multivariable model including all potential predictors shown in the figure. A higher chi-square value corresponds to a higher independent prognostic value. ASA PS, ASA performance score; RPD, robot-assisted pancreatoduodenectomy; OPD, open pancreatoduodenectomy; OS, overall survival.

In the multivariable analysis, the strongest prognostic factors for OS were positive lymph node ratio (χ^2^ = 161.8, *P* < 0.001), CA19.9 level before treatment (χ^2^ = 24.7, *P* < 0.001), differentiation grade (χ^2^ = 23.7, *P* < 0.001), and age at diagnosis (χ^2^ = 8.9, *P* = 0.012), whereas tumour diameter (χ^2^ = 7.1, *P* = 0.068), ASA score (χ^2^ = 3.5, *P* = 0.063), CCI score (χ^2^ = 3.5, *P* = 0.063), NAT (χ^2^ = 2.8, *P* = 0.094), mGPS (χ^2^ = 1.9, *P* = 0.397), diabetes mellitus (χ^2^ = 0.4, *P* = 0.517), sex (χ^2^ = 0.3, *P* = 0.617), and surgical technique (RPD *versus* OPD) were not significantly prognostic for OS (χ^2^ = 0.2, *P* = 0.663) (*Fig. 2*).

**Fig. 3:**
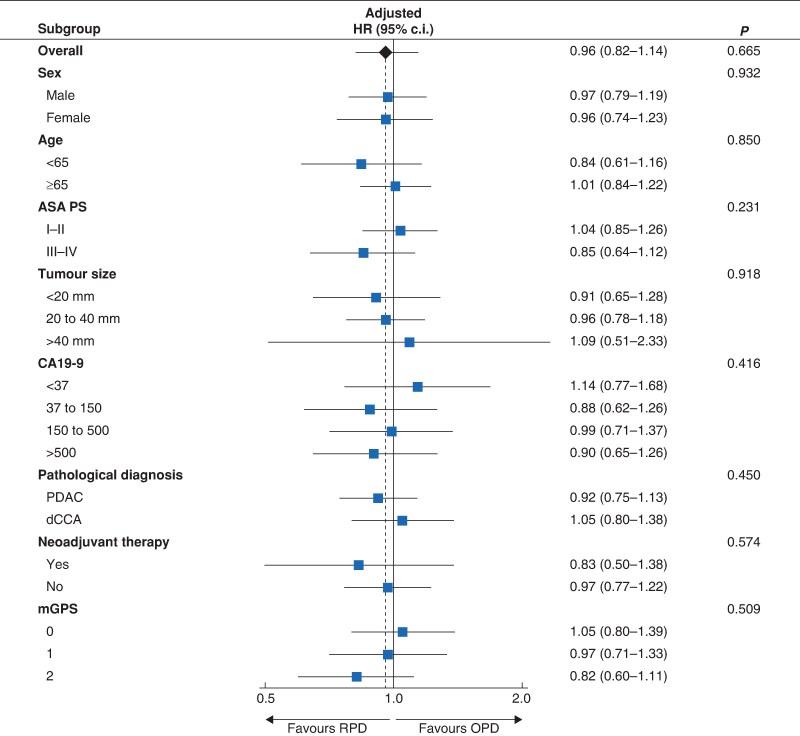
Forest plot of the impact of the surgical approach on overall survival per subgroup The forest plot of surgical approach effect (point estimate) on overall survival per subgroup with 95% confidence interval (error bar). Size of the squares are proportional to the number of patients. The interrupted line represents the average treatment effect. RPD, robot-assisted pancreatoduodenectomy; OPD, open pancreatoduodenectomy; PDAC, pancreatic ductal adenocarcinoma; DCC, distal cholangiocarcinoma.

The interaction analysis showed that the impact of surgical approach on OS did not differ between subgroups regarding sex (*P* = 0.932), age (*P* = 0.850), ASA score (*P* = 0.231), tumour size (*P* = 0.918), CA19.9 level (*P* = 0.416), pathological diagnosis (*P* = 0.450), NAT (*P* = 0.574), and mGPS (*P* = 0.509) (*Fig. 3*).

## Discussion

This nationwide retrospective cohort study including patients after pancreatoduodenectomy in an 8-year period found a comparable OS between RPD and OPD for upfront resectable cancer in the pancreatic head without vascular involvement on preoperative imaging. The surgical approach was not an independent predictor for survival and no specific patient subgroups showed a significant OS benefit from RPD or OPD. The R0-resection rate and positive lymph node ratio did not differ significantly between RPD and OPD. Administration, time to start, and completion of adjuvant therapy were also comparable between groups.

The current study found a comparable OS after RPD and OPD for patients with cancer in the pancreatic head. In accordance with these results, previous studies showed no significant differences in OS between RPD and OPD^6,46–51^. However, two other studies did find a difference in OS. A retrospective study from a high-volume centre in Taiwan including 304 RPD and 172 OPD in patients with periampullary cancers reported an improved OS in favour of RPD (median 20 *versus* 18 months, *P* < 0.001)^52^. However, this study included all periampullary cancers with relatively few PDAC (85 RPD *versus* 81 OPD) and DCC (16 RPD *versus* 10 OPD). Similarly, a recent propensity-score matched study from Shanghai, China including 103 RPD and 206 OPD after the learning curve demonstrated that RPD was an independent predictor for improved OS in patients with PDAC^19^. However, both above-mentioned studies did not investigate oncological outcome in a nationwide multicentre setting. The present study showed a considerable numeric difference in 5-year survival rate after surgery between patients with PDAC with *versus* without NAT (35.2% *versus* 20.2% after RPD and 30.3% *versus* 18.8% after OPD). The impact of NAT on OS in upfront resectable PDAC is currently being investigating in the PREOPANC-3 randomized trial^35^ .

The present study found a comparable R0-resection rate and positive lymph node ratio between RPD and OPD. The EUROPA randomized trial from Heidelberg, Germany investigated short-term oncological outcome in 29 RPD *versus* 33 OPD (including 34 patients with malignant disease) and reported a comparable R0-resection rate (43.8% *versus* 50.0%, *P* = 0.154) and lymph node yield (median 29 *versus* 26, *P* = 0.536) between groups^17^. A recent randomized trial from Beijing, China with 81 RPD and 80 OPD (including 120 patients with malignant disease) reported a similar lymph node yield (median 13 *versus* 13, *P* = 0.36) with comparable and remarkably high R0-resection rates in both groups (89% *versus* 88%, *P* = 0.79)^17,18^. It is important to note that the definition of ‘R0-resection’ differs between countries. The Royal College of Pathologists definition includes the tumour margin distance from the vascular groove, anterior surface and non-uncinate posterior surface, in contrast to the College of American Pathologists. Therefore, comparison of R0-resection rate between countries is difficult. In the current study, a median of 15 lymph nodes was resected with RPD and 16 with OPD. The clinical relevance of median one resected lymph node difference is negligible. The ISGPS advises a minimum of 15 resected lymph nodes to ensure adequate pathological staging of the disease^30,39^.

Adjuvant chemotherapy for PDAC was administered in 58.2% of patients after RPD and 58.7% after OPD with a comparable time to start (median 54 *versus* 58 days). The Beijing trial demonstrated a shorter time to start of adjuvant therapy after RPD than OPD (median 42 *versus* 49 days). A large retrospective study from the United States including 626 RPD and 17 205 OPD from the National Cancer Database did not find a significant difference between groups^18,53^. The variation across countries may be attributable to variations in healthcare logistics and socioeconomic factors^54^ . Ideally, research should focus on determining when patients are fit for adjuvant therapy, based on oncologist assessment, rather than the actual date of initiation. Furthermore, time to adjuvant chemotherapy in the RPD group might improve as surgical and oncological experience increases. A study of the National Cancer Database in the United States found that conversion of RPD to OPD was associated with a longer time to adjuvant chemotherapy^53^. As experience increases, conversion rates in RPD will decrease, potentially shortening the time to adjuvant chemotherapy. In patients with adjuvant chemotherapy for PDAC, more dose reductions were observed after RPD than OPD. In 2018, RPD was implemented following the LAELAPS-3 training programme^55^, whereas this cohort included patients undergoing OPD from 2016 onwards. Development in adjuvant chemotherapy may have had an impact on the OS after these two approaches. This potential time bias was minimized in the multivariable analysis and the sensitivity analysis excluding procedures before 2018, which had no impact on OS.

Finally, even though vascular involvement was not present on preoperative imaging, the vascular resection rate in RPD and OPD was 5.9% and 13.9% respectively. Disease progression with vascular contact since the performance of the last imaging is a likely explanation for this finding^1^. Also, fully excluding the possibility of any vascular contact in patients with PDAC and DCC on cross-sectional imaging can be challenging^56^.

The findings of the present study should be interpreted considering several limitations. First, the retrospective study design resulted in some missing data. Preoperative BMI was reported in only 2% of patients, so OPD patients could not be selected based on BMI. To address potential confounding related to preoperative physical status, the mGPS was included in the multivariable analysis. Second, the retrospective study design may have introduced treatment selection bias. For example, more patients in the RPD group received NAT compared to OPD. The six-month ‘immortal’ time bias associated with NAT may have led to an underestimation of survival after RPD, whereas the fact that patients who had progression during NAT were not included in this cohort could have led to an overestimation of survival after RPD. The difference in receipt of NAT may be explained by the fact that neoadjuvant therapy was only administered in patients with PDAC who participated in the PREOPANC trials as the current Dutch guidelines recommend upfront resection in resectable PDAC and DCC^35,36,57^. Only seven high-volume centres in the Netherlands performed RPD with a relatively high inclusion rate in the PREOPANC trials^35^. In addition, the present study did not correct for potential centre-specific bias as these data were not available. Another example of potential selection bias was that fewer vascular resections were performed with RPD. Third, we included both patients with PDAC and DCC as ‘cancer in the pancreatic head’. The rather comparable 1-, 3-, and 5-year survival rates support this choice. For this reason, only these two types of cancer are included in the ongoing DIPLOMA-2×2 randomized trial (ISRCTN27483786). Fourth, recurrence-free survival could not be assessed due to limited data availability in NCR, resulting in too few events in the RPD group. Fifth, before 2019, tumour differentiation was classified according to the fourth edition of the WHO Classification of Tumours in the Digestive System, based on gland formation percentage. The updated classification (fifth) considers even a small component of poor differentiation sufficient to classify the tumour as poorly differentiated. Therefore, diagnoses before 2019 may have been under classified in terms of poor differentiation, potentially affecting the comparability of tumour grading over time. Sixth, all patients undergoing RPD and OPD were included, including patients treated during the RPD learning curve. This study represents the earliest assessment of oncological outcome after RPD, compared to the extensive experience with OPD, potentially underestimating the effect of RPD. On the other hand, residual selection bias could have favoured survival after RPD, although potential selection bias was minimized in the multivariable analysis. Multivariable analysis was considered sufficient due to the high number of events, in contrast to propensity-score matching, which would have excluded patients and decreased the study’s power. In summary, the oncological safety of RPD cannot be conclusively determined. To what extent these outcomes are explained by confounding should be assessed by future randomized studies such as the ongoing DIPLOMA-2×2 trial (ISRCTN27483786).

## Supplementary Material

znaf153_Supplementary_Data

## Data Availability

The data of the present study are available on request from the corresponding author. Due to privacy and ethical regulations, the data are not publicly available.
