# Supplementary material for: Oncological outcome after robot-assisted versus open pancreatoduodenectomy for upfront resectable cancer in the pancreatic head: a nationwide cohort analysis
Source: Br J Surg. 2025 Nov 28;112(11):znaf153. doi: 10.1093/bjs/znaf153 (PMC12661940; doi:10.1093/bjs/znaf153)
Supplement: znaf153_Supplementary_Data [file znaf153_supplementary_data.docx]

**Oncological o****utcome after robot-assisted versus open pancreatoduodenectomy for upfront resectable cancer in the pancreatic head: a nationwide analysis**

Julia E. Menso*^1,2^, Caro L. Bruna*^1,2^, Mahsoem Ali^2,3^, Bert Bonsing^4^, Koop Bosscha^5^, Lodewijk A.A. Brosens^6^, Olivier R. Busch^1,2^, A. Stijn L.P. Crobach^7^, Freek Daams^2,3^, Wouter Derksen^8^, Maxime J.L. Dewulf^9^, Michail Doukas^10^, Arantza Fariña Sarasqueta^2,11^, Sebastiaan Festen^12^, Mohammad Abu Hilal^1,13,14^, Ignace H.J.T. de Hingh^15^, Marjolein Y.V. Homs^16^, Geert Kazemier^2,3^, Daan J. Lips^17^, Misha D.P. Luyer^15^, Vincent E. de Meijer^18^, J. Sven D. Mieog^4^, Wouter W. te Riele^8^, Hjalmar C. van Santvoort^8^, George P. van der Schelling^19^, Martijn Stommel^20^, Joanne Verheij^2,10^, Roeland F. de Wilde^21^, Johanna W. Wilmink^2,22^, I. Quintus Molenaar^8^, Bas Groot Koerkamp*^21^, Lydia G. van der Geest*^23^, Marc G. Besselink*^1,2^, on behalf of the Dutch Pancreatic Cancer Group

**Shared first/senior authorship*

**Affiliations**

1 Amsterdam UMC, location University of Amsterdam, Department of Surgery, Amsterdam, The Netherlands

2 Cancer Center Amsterdam, Amsterdam, The Netherlands

3 Amsterdam UMC, location Vrije Universiteit, Department of Surgery, Amsterdam, The Netherlands

4 Leiden University Medical Center, Department of Surgery, Leiden, The Netherlands

5 Jeroen Bosch Hospital, Department of Surgery, ‘s-Hertogenbosch, The Netherlands

6 Regional Academic Cancer Center Utrecht (RAKU), UMC Utrecht, Department of Pathology, Utrecht, The Netherlands

7 Leiden University Medical Center, Department of Pathology, Leiden, The Netherlands

8 Regional Academic Cancer Center Utrecht (RAKU), UMC Utrecht, Department of Surgery, Utrecht, The Netherlands

9 Maastricht University Medical Center, Department of Surgery, Maastricht, The Netherlands

10 Erasmus MC, Department of Pathology, Rotterdam, The Netherlands

11 Amsterdam UMC, location University of Amsterdam, Department of Pathology, Amsterdam, The Netherlands

12 OLVG, Department of Surgery, Amsterdam, The Netherlands

13 Department of Surgery, School of Medicine, University of Jordan, Amman 11942, Jordan

14 Department of Surgery, University Hospital Southampton NHS Foundation Trust, Southampton, United Kingdom

15 Catharina Hospital, Department of Surgery, Eindhoven, The Netherlands

16 Erasmus MC Cancer Institute, Department of Medical Oncology, Rotterdam, The Netherlands

17 Medisch Spectrum Twente, Department of Surgery, Enschede, The Netherlands

18 University of Groningen and University Medical Center Groningen, Department of Surgery, Groningen, The Netherlands

29 Amphia Hospital, Department of Surgery, Breda, The Netherlands

20 Radboud University Medical Center, Department of Surgery, Nijmegen, The Netherlands

21 Erasmus MC Cancer Institute, Department of Surgery, Rotterdam, The Netherlands

22 Amsterdam UMC, location University of Amsterdam, Department of Medical Oncology, Amsterdam, The Netherlands

23 Netherlands Comprehensive Cancer Organisation (IKNL), Department of Research and Development, Utrecht, The Netherlands

**Correspondence:**

Marc Besselink, MD MSc PhD, [m.g.besselink@amsterdamUMC.nl](mailto:m.g.besselink@amsterdamUMC.nl)

Amsterdam UMC, location University of Amsterdam,

Department of Surgery, Cancer Center Amsterdam

De Boelelaan 1117 (ZH-7F), 1081 HV Amsterdam, The Netherlands.

**Supplementary Materials - Index**

| **Supplementary Figures and Tables** |  |
| --- | --- |
| Supplementary table 1: STROBE checklist | *page 4-5* |
| Supplementary table 2: Eligibility criteria according to the National Cancer Registry definitions | *page 6* |
| Supplementary table 3: Unadjusted survival of patients undergoing robot-assisted versus open pancreatoduodenectomy | *page 7* |
| Supplementary table 4: Adjusted survival of patients with pancreatic ductal adenocarcinoma undergoing robot-assisted versus open pancreatoduodenectomy with and without neoadjuvant therapy | *page 8* |
| Supplementary figure 1: Annual procedure count for robot-assisted and open pancreatoduodenectomy for PDAC and DCC | *page 9* |
| Supplementary figure 2: Kaplan-Meier curve of the unadjusted overall survival of patients undergoing robot-assisted versus open pancreatoduodenectomy | *page 10* |
|  |  |

# Supplementary table 1: STROBE checklist

|  | Item No | Recommendation | Location where item is reported |
| --- | --- | --- | --- |
| **Title and abstract** | 1 | (*a*) Indicate the study’s design with a commonly used term in the title or the abstract | Page 1-2 |
|  |  | (*b*) Provide in the abstract an informative and balanced summary of what was done and what was found | Page 3 |
| Introduction | | | |
| Background/rationale | 2 | Explain the scientific background and rationale for the investigation being reported | Page 4 |
| Objectives | 3 | State specific objectives, including any prespecified hypotheses | Page 4 |
| Methods | | | |
| Study design | 4 | Present key elements of study design early in the paper | Page 5 |
| Setting | 5 | Describe the setting, locations, and relevant dates, including periods of recruitment, exposure, follow-up, and data collection | Page 5 |
| Participants | 6 | (*a*) Give the eligibility criteria, and the sources and methods of selection of participants. Describe methods of follow-up | Page 5-6 |
|  |  | (*b*) For matched studies, give matching criteria and number of exposed and unexposed | Not applicable |
| Variables | 7 | Clearly define all outcomes, exposures, predictors, potential confounders, and effect modifiers. Give diagnostic criteria, if applicable | Page 6-8 |
| Data sources/ measurement | 8 | For each variable of interest, give sources of data and details of methods of assessment (measurement). Describe comparability of assessment methods if there is more than one group | Page 6-8 |
| Bias | 9 | Describe any efforts to address potential sources of bias | Page 8-10 |
| Study size | 10 | Explain how the study size was arrived at | Page 5 |
| Quantitative variables | 11 | Explain how quantitative variables were handled in the analyses. If applicable, describe which groupings were chosen and why | Page 8-10 |
| Statistical methods | 12 | (*a*) Describe all statistical methods, including those used to control for confounding | Page 8-10 |
|  |  | (*b*) Describe any methods used to examine subgroups and interactions | Page 8-10 |
|  |  | (*c*) Explain how missing data were addressed | Page 8-10 |
|  |  | (*d*) If applicable, explain how loss to follow-up was addressed | Not applicable |
|  |  | (*e*) Describe any sensitivity analyses | Page 8-10 |
| Results | | | |
| Participants | 13 | (a) Report numbers of individuals at each stage of study—eg numbers potentially eligible, examined for eligibility, confirmed eligible, included in the study, completing follow-up, and analysed | Page 11 |
|  |  | (b) Give reasons for non-participation at each stage | Not applicable |
|  |  | (c) Consider use of a flow diagram | Not applicable |
| Descriptive data | 14 | (a) Give characteristics of study participants (eg demographic, clinical, social) and information on exposures and potential confounders | Page 11 |
|  |  | (b) Indicate number of participants with missing data for each variable of interest | Not applicable |
|  |  | (c) Summarise follow-up time (eg, average and total amount) | Page 12 |
| Outcome data | 15 | Report numbers of outcome events or summary measures over time | Page 12-13 |
| Main results | 16 | (*a*) Give unadjusted estimates and, if applicable, confounder-adjusted estimates and their precision (eg, 95% confidence interval). Make clear which confounders were adjusted for and why they were included | Page 12-13 |
|  |  | (*b*) Report category boundaries when continuous variables were categorized | Page 8-10 |
|  |  | (*c*) If relevant, consider translating estimates of relative risk into absolute risk for a meaningful time period | Not applicable |
| Other analyses | 17 | Report other analyses done—eg analyses of subgroups and interactions, and sensitivity analyses | Page 12-13 |
| Discussion | | | |
| Key results | 18 | Summarise key results with reference to study objectives | Page 14 |
| Limitations | 19 | Discuss limitations of the study, taking into account sources of potential bias or imprecision. Discuss both direction and magnitude of any potential bias | Page 16-17 |
| Interpretation | 20 | Give a cautious overall interpretation of results considering objectives, limitations, multiplicity of analyses, results from similar studies, and other relevant evidence | Page 14-17 |
| Generalisability | 21 | Discuss the generalisability (external validity) of the study results | Page 16-17 |
| Other information | | | |
| Funding | 22 | Give the source of funding and the role of the funders for the present study and, if applicable, for the original study on which the present article is based | Page 23 |

Legend supplementary table 1: The STROBE checklist. Reference: Vandenbroucke, J.P., et al., *Strengthening the Reporting of Observational Studies in Epidemiology (STROBE): explanation and elaboration.* Int J Surg, 2014. **12**(12): p. 1500-24.

# Supplementary table 2. Eligibility criteria according to the National Cancer Registry definitions

| **Eligibility criteria** | **Description** |
| --- | --- |
| Time frame | Date of diagnosis between 01-01-2016 and 31-12-2023 |
| Tumor group | Hepato-Pancreato-Biliary |
| Topography | According to the International Classification for Oncology (4^th^ edition):  C24.2 – Distal bile duct  C25 – Pancreas (excluding C25.4) |
| Tumor behavior | Invasive |
| Morphology | Morphology codes (including adenocarcinoma in precursor lesions):  8003, 8012, 8020, 8021, 8035, 8070, 8140, 8144, 8160, 8163, 8211, 8260, 8310, 8450, 8453, 8470, 8480, 8481, 8490, 8500, 8503, 8510, 8560 |
| Age | ≥18 years |
| Sex | Male and female |
| Level | Nationwide |
| Surgery type | Whipple  PPPD  PRPD  ‘No further specified’ if tumor location ‘distal bile duct’ or ‘pancreatic head’ |
| Surgical technique | Robot-assisted surgery  Open surgery |
| Vascular involvement (venous or arterial) | No |

Legend Supplementary table 2: Details of the eligibility criteria according to the definitions of the National Cancer Registry. PD, pancreatoduodenectomy; PPPD, pylorus-preserving pancreatoduodenectomy; PRPD, pylorus-resecting pancreatoduodenectomy.

| **Survival (unadjusted)** | **RPD (n=375)** | **OPD (n=1,300)** | **HR (95% CI)** | **P-value** |
| --- | --- | --- | --- | --- |
| Overall survival (months) | 24 (22–29) | 23 (21–24) | 0.93 (0.79–1.08) | 0.312 |
| 1-year survival | 72.8% (68.2–77.7) | 72.5% (70.0–75.0) | N/A | N/A |
| 3-year survival | 35.9% (30.4–42.5) | 33.5% (30.7–36.5) | N/A | N/A |
| 5-year survival | 27.3% (21.3–35.0) | 21.7% (19.1–24.7) | N/A | N/A |

# Supplementary table 3. Unadjusted survival of patients undergoing robot-assisted versus open pancreatoduodenectomy

Legend supplementary table 3: Values are presented in medians with interquartile ranges (IQR) or percentages with 95% confidence intervals. RPD, robot-assisted pancreatoduodenectomy; OPD, open pancreatoduodenectomy; HR, Hazard ratio; CI, confidence interval.

| **Survival (adjusted)** | **RPD (n=375)** | **OPD (n=1,300)** | **HR (95% CI)** | **P-value** |
| --- | --- | --- | --- | --- |
| Survival PDAC with NAT, months | 26 (14–NE) | 21 (15–33) | 0.80 (0.43–1.47) | 0.477 |
| 1-year survival | 74.5% (63.2–83.2) | 70.7% (63.4–77.0) | N/A | N/A |
| 3-year survival | 43.4% (29.3–58.6) | 38.3% (29.7–47.8) | N/A | N/A |
| 5-year survival | 35.2% (21.3-52.1) | 30.3% (20.9–41.6) | N/A | N/A |
| Survival PDAC without NAT, months | 23 (18–29) | 22 (19–24) | 0.96 (0.77–1.21) | 0.743 |
| 1-year survival | 71.8% (66.9–76.3) | 70.5% (67.9–73.1) | N/A | N/A |
| 3-year survival | 32.0% (26.0–38.7) | 30.3% (27.2–33.6) | N/A | N/A |
| 5-year survival | 20.2% (15.1–26.4) | 18.8% (15.8–22.1) | N/A | N/A |

# Supplementary table 4. Adjusted survival of patients with pancreatic ductal adenocarcinoma undergoing robot-assisted versus open pancreatoduodenectomy with and without neoadjuvant therapy

Legend supplementary table 4: Values are presented in medians with interquartile ranges (IQR) or percentage with 95% confidence intervals (CI). RPD, robot-assisted pancreatoduodenectomy; OPD, open pancreatoduodenectomy; HR, Hazard ratio; CI, confidence interval; PDAC, pancreatic ductal adenocarcinoma; NAT, neoadjuvant therapy.

# Supplementary figure 1. Annual procedure count for robot-assisted and open pancreatoduodenectomy for PDAC and DCC


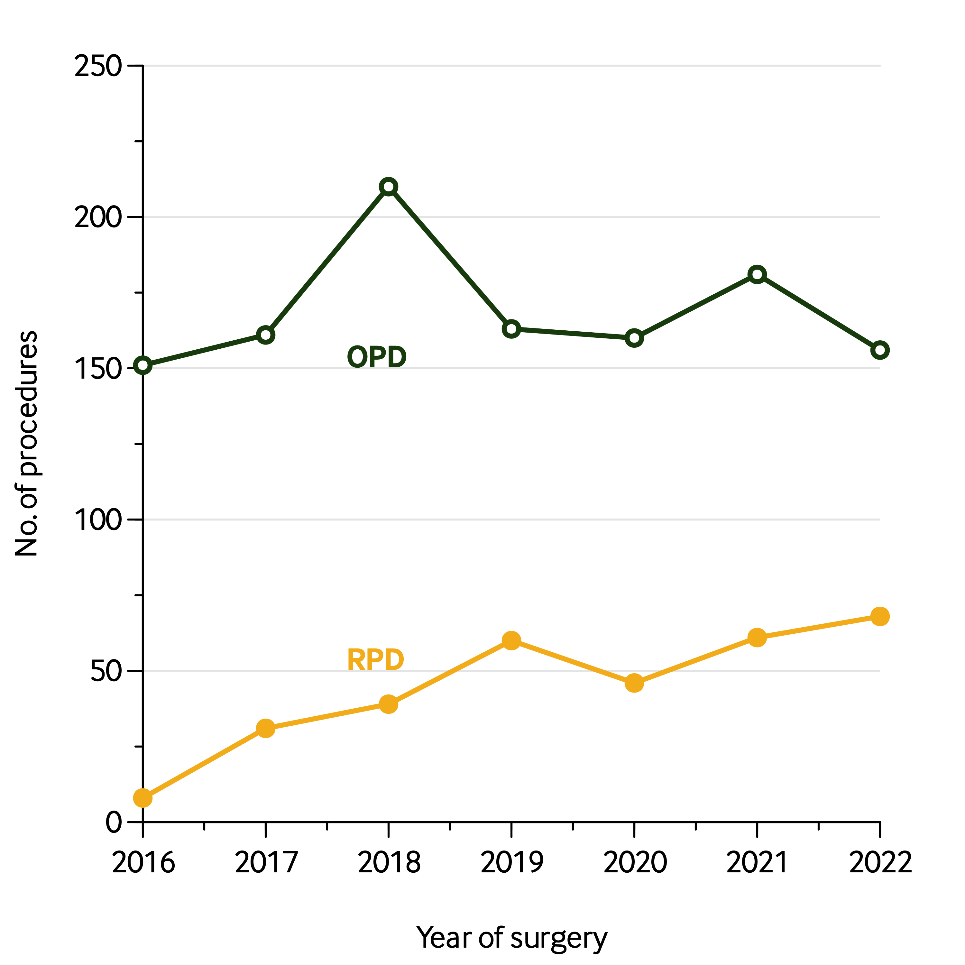


Legend supplementary figure 1: Values are number of procedures (y-axis) per year (x-axis) for OPD (upper) and RPD (lower). RPD, robot-assisted pancreatoduodenectomy; OPD, open pancreatoduodenectomy.

# Supplementary figure 2. Kaplan-Meier curve of the unadjusted overall survival of patients undergoing robot-assisted versus open pancreatoduodenectomy


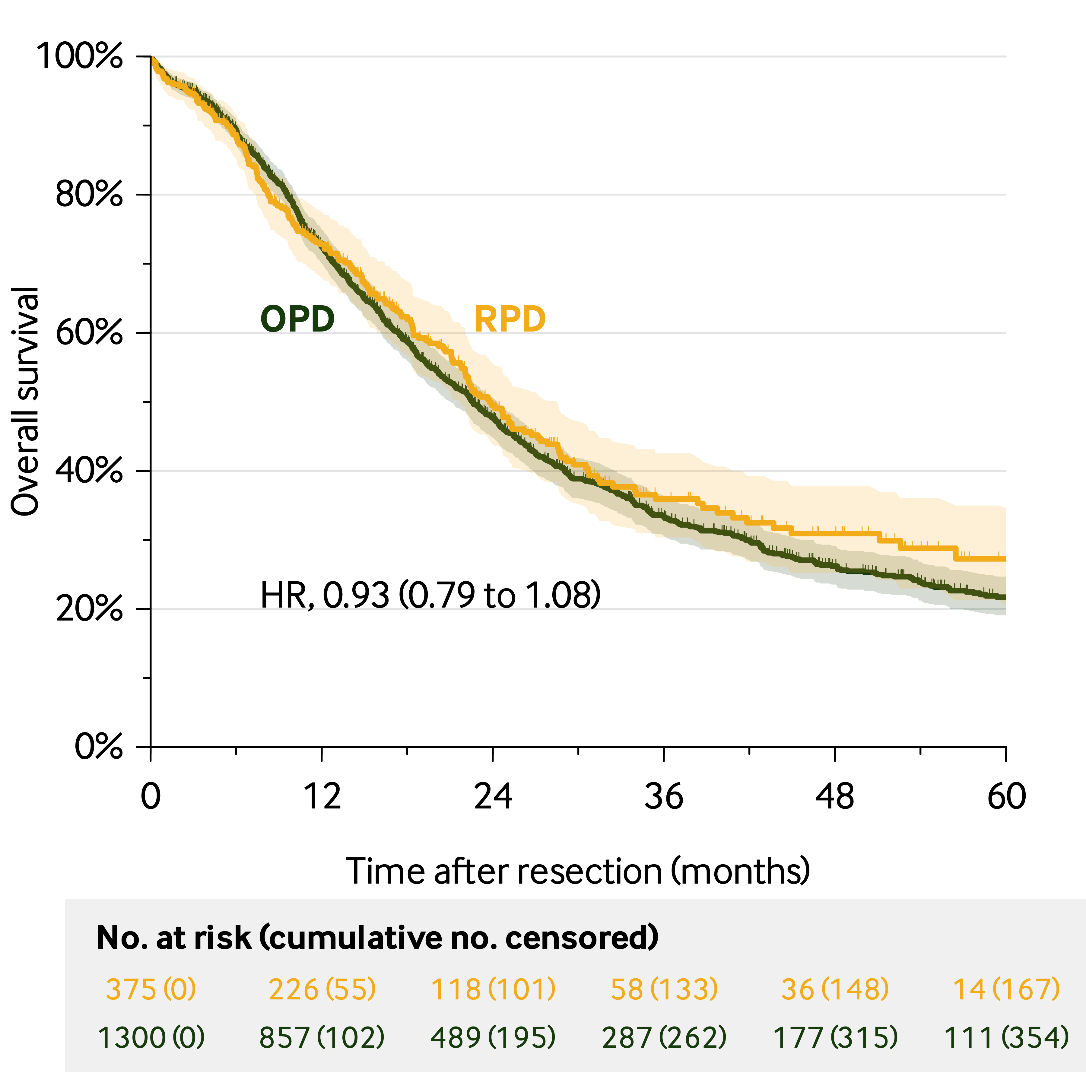


Legend Supplementary figure 2: Kaplan-Meier curve of the unadjusted overall survival after robot-assisted (yellow) and open pancreatoduodenectomy (green). RPD, robot-assisted pancreatoduodenectomy; OPD, open pancreatoduodenectomy; HR, hazard ratio.
